# The pterional approach for the surgical resection of an orbital cholesteatoma: a case report

**DOI:** 10.1186/s13256-020-02579-3

**Published:** 2021-01-22

**Authors:** Parménides Guadarrama-Ortiz, José Alberto Choreño-Parra, Francisco Javier Pacheco-Sánchez, Alberto Iván Rodríguez-Nava, Gabriela García-Quintero, Patricia Emilia Rodríguez-Muñoz, Carlos Sánchez-Garibay, Manuel Ángeles-Castellanos

**Affiliations:** 1 Centro Especializado en Neurocirugía y Neurociencias Mexico (CENNM), Mexico City, Mexico; 2 Escuela Nacional de Medicina y Homeopatía, Instituto Politécnico Nacional, Mexico City, Mexico; 3 Departamento de Neuropatología, Instituto Nacional de Neurología y Neurocirugía “Manuel Velasco Suárez”, Mexico City, Mexico; 4 Departamento de Anatomía, Facultad de Medicina, Universidad Nacional Autónoma de México, Mexico City, Mexico

**Keywords:** Orbital tumors, Cholesteatoma, Cholesterol granuloma, Pterional approach, Ocular surgery, Neurosurgery

## Abstract

**Background:**

Cholesteatomas are benign tumors mainly composed of cholesterol crystals that rarely arise within the orbit. However, orbital cholesteatomas require a complete surgical resection due to their recidivating potential. Transcranial approaches offering a broad surgical exposure of the orbital cavity have been scarcely used for the management of these tumors. Here, we provide evidence of the benefits of the pterional craniotomy for the surgical resection of orbital tumors by sharing our experience in the surgical management of a cholesteatoma of the superotemporal orbital wall.

**Case presentation:**

A 45-year-old Hispanic man with a 2-year history of progressive proptosis of the left eye attended to our center complaining of diplopia and migraine. At his arrival, physical examination revealed ptosis, palpebral edema, and exophthalmos of the left eye, as well as the abolishment of the ipsilateral photomotor and consensual responses. Fundoscopy showed mild optic atrophy, whereas a T2-weighted magnetic resonance imaging (MRI) of the head showed a hyperintense mass arising at the superotemporal wall of the orbit that was displacing the eyeball. The tumor was resected using a pterional craniotomy without postoperative complications. The histopathological analysis of the tumor revealed a cholesteatoma. The patient recovered the functionality of the left eye with no visual sensitive deficits nor tumor recurrence 1 year after the surgery.

**Conclusions:**

Our results support the use of the pterional craniotomy as a safe procedure for the surgical resection of cholesteatomas arising at the superotemporal walls of the orbit, with low postoperative morbidity.

## Background

Different inflammatory, vascular, cystic, and neoplastic lesions have their origin at the orbit. Among these, mesenchymal and lymphoproliferative tumors account for most cases of orbital lesions, although other neurogenic, lacrimal, and metastatic tumors can also affect this location [1, 2]. Cholesteatomas are benign tumors mainly composed of cholesterol crystals that rarely arise within the orbit [3]. Depending on the presence or absence of an epithelial component, these lesions are classified as "true cholesteatomas" and "cholesterol granulomas," respectively. Independently of their histological characteristics, both entities require a complete surgical resection due to the recidivating nature and potential of malignant progression of cholesteatomas, as well as the extension and erosive capacity of cholesterol granulomas [3, 4].

Distinct approaches have been employed to access orbital tumors, most of them requiring extensive bony removal that hinders the total resection of complex masses without risk of postoperative complications and aesthetic consequences [5]. Conversely, transcranial approaches are less frequently advocated for the resection of orbital tumors. The pterional craniotomy is the most common surgical approach used in neurosurgery to access lesions arising along the anterior and middle skull base [6, 7], which also provides a good exposure of the orbit through its roof and superotemporal wall [8]. Despite this, the pterional approach has been scarcely used to access orbital tumors. Hence, the usefulness of this approach for the management of complex and potentially recurrent orbital tumors, such as cholesteatomas, is currently unknown.

In the current report, we provide evidence of the safety and advantages of the pterional approach for the surgical management of a patient with a cholesteatoma arising at the superolateral orbital wall. Our findings indicate that the pterional craniotomy is a suitable procedure for the surgical resection of orbital cholesteatomas with low operative morbidity.

## Case presentation

**Fig. 1.**
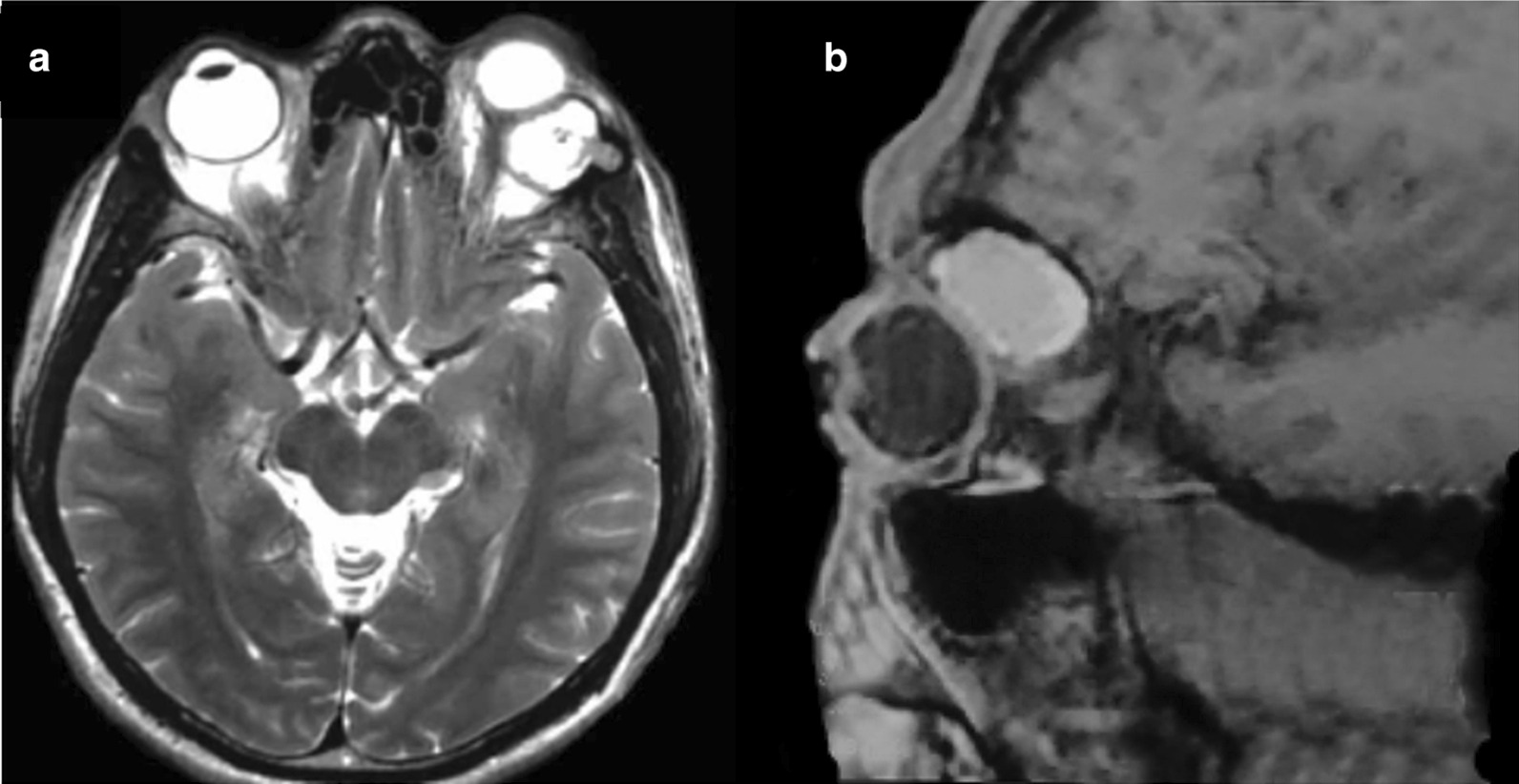
Preoperative clinical findings in a male patient with an orbital cholesteatoma. **a** Transversal T2-weighted magnetic resonance images (MRI) of the brain showing an hyperintense mass located at the superolateral orbital wall of the left eye. **b** Sagittal T2-weighted MRI which reveals the anterior displacement of the left eye and the invasion of the frontal bone caused by the tumor

A 45-year-old Hispanic man with a 2-year history of progressive proptosis of the left eye attended to our center complaining of diplopia and hemicranial migraine. He was previously diagnosed with hyperthyroidism receiving thyrostatic treatment with thiamazole for 6 months without clinical improvement. His past medical history was not relevant, and he denied previous ocular trauma. At his arrival, physical examination revealed ptosis, palpebral edema, and exophthalmos of the left eye, as well as the abolishment of the ipsilateral photomotor and consensual responses, but conserving the corneal reflex. Furthermore, the patient presented an evident limitation for the abduction and supraduction of the affected eye, whereas fundoscopy showed mild optic atrophy. The exploration of the right eye did not reveal any abnormality, whereas the neurological examination showed normal cognitive function with no focal neurologic deficits. A T2-weighted magnetic resonance imaging (MRI) of the head showed a hyperintense mass arising at the superotemporal wall of the left orbit that was displacing the eyeball, invading the frontal bone, and causing inflammation of the adjacent dura mater (Fig. 1a, c).

**Fig. 2.**
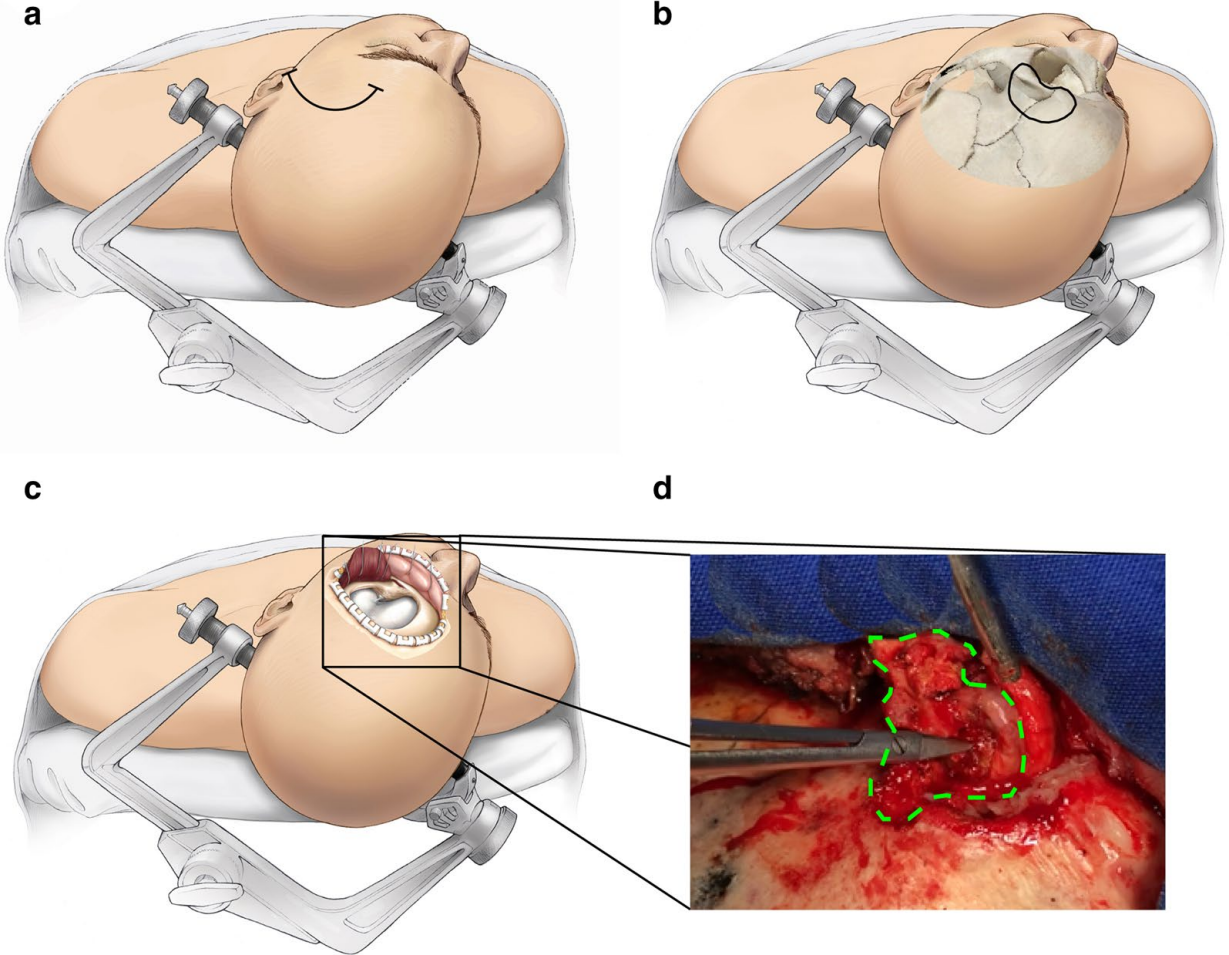
Surgical technique of the pterional approach for the resection of a left orbital cholesteatoma. **a** Skin incision. **b** Dissection of the fascia of the temporalis muscle and exposure of the temporal fossa. **c** Osteoplastic removal of the superolateral orbital wall. **d** Macroscopic aspect of the resected tumo

**Fig. 3.**
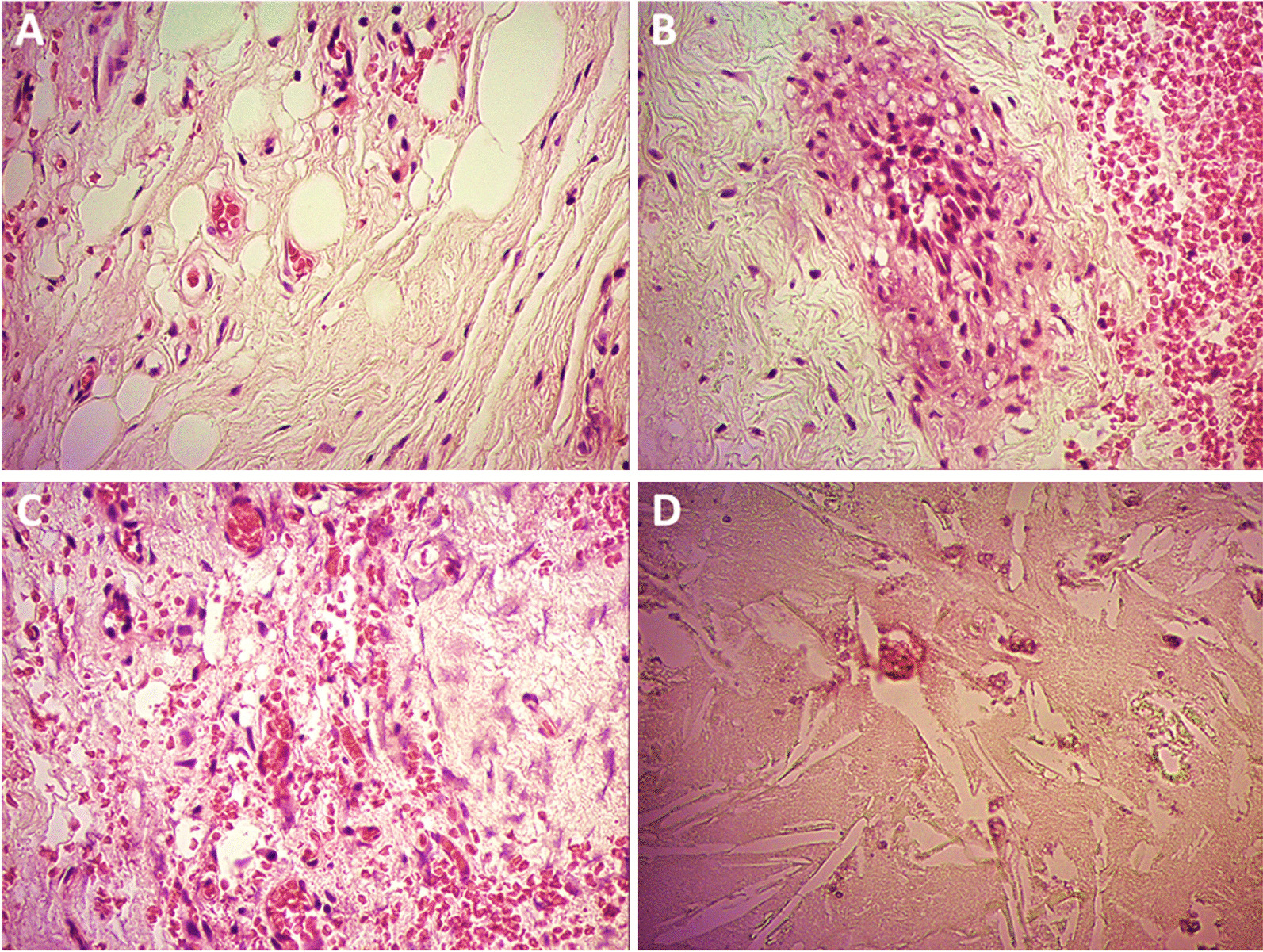
Histological characteristics of the orbital cholesteatoma. The histological analysis of the resected tumor showed classic components of a cholesteatoma such as fibrosis (**a**), hematic content and mixed inflammatory infiltrate (**b**, **c**), as well as cholesterol crystals (**d**). The absence of an epithelial component indicates that this tumor was indeed a cholesterol granuloma

**Fig. 4.**
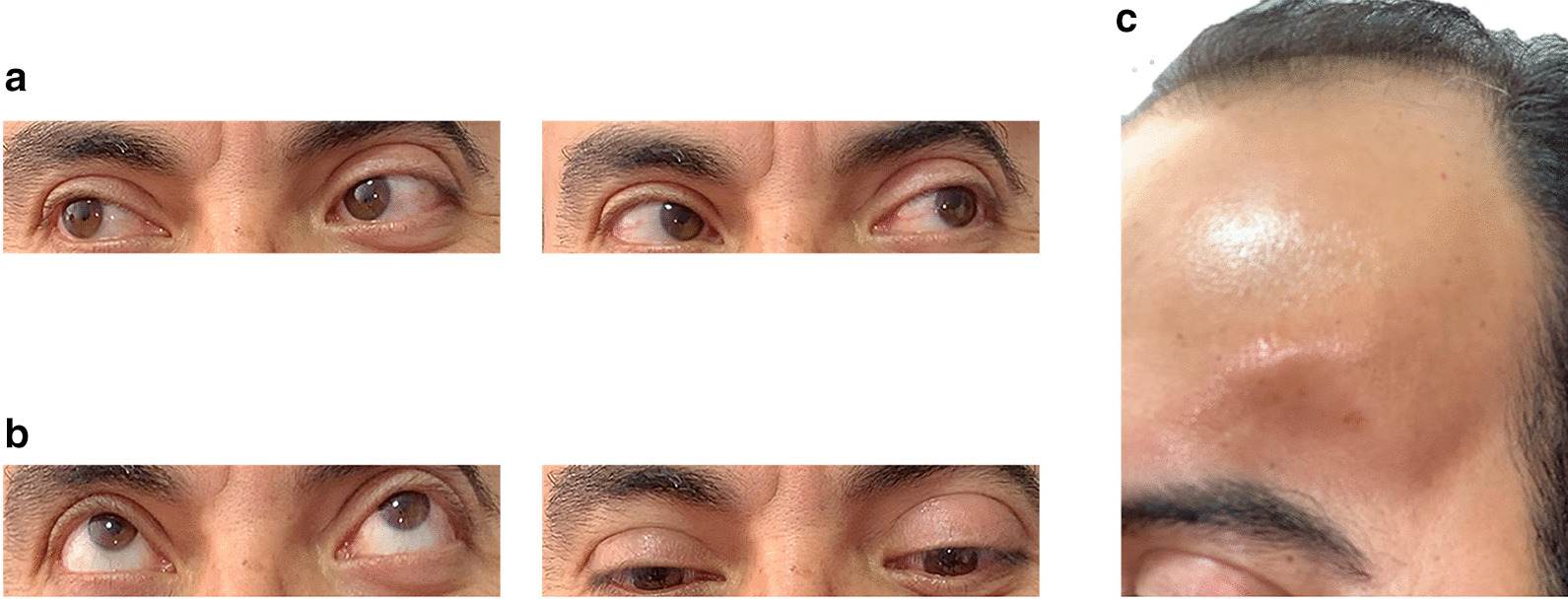
Postoperative clinical findings after the surgical removal of an orbital cholesteatoma using the pterional approach. A year after surgery the patient recovered sensorial functioning, as well as lateral (**a**), and vertical (**b**) movements of the left eye, remaining only with minimal ptosis and exophthalmos. The aesthetical benefits of the pterional approach are evident, as the scar of the skin incision is hidden within the hair line and does not affect the forehead and face (**c**)

Due to the extension of the tumor, we decided on its surgical resection using a pterional approach. The skin incision and dissection of the temporalis muscle fascia were performed as described before [6, 7], in order to expose the temporal fossa and the orbital roof (Fig. 2a–c). To get access to the orbit, we performed the osteoplastic removal of the superolateral orbital wall (formed by the greater sphenoidal wing) using a surgical drill, with no need for opening the dura mater (Fig. 2c) [8]. This technical modification allowed us to completely resect a well-defined mass of hematic brown color, invading the frontal bone to the level of the frontozygomatic joint and the orbital floor, with minimal risk of lesioning intradural and orbital structures (Fig. 2d). The histopathological analysis of the resected tumor indicated the presence of abundant fibrous tissue, cholesterol crystals, coagulated hematic content, and a mixed inflammatory infiltrate with a predominance of lymphocytes and foamy histiocytes (Fig. 3), compatible with the diagnosis of cholesteatoma. The patient recovered the functionality of the left eye with no deficits in the visual acuity nor diplopy (Fig. 4a–c), although he remained with a low degree of ptosis. Finally, a second MRI was performed, which showed no residual tumor 1-year after the surgery.

## Discussion and conclusions

Cholesteatomas and cholesterol granulomas are benign lesions of presumed inflammatory origin that rarely affect the orbit [3]. However, due to their invasive, recidivating, and progressive characteristics, their management requires a complete resection from their bony bed [4]. The optimal surgical approach to these tumors is unknown, and as for other orbital lesions, their management has been based on their extension, growth rate, and localization within the orbit. Most cases reported before have been treated using anterior and lateral orbitotomy approaches, whereas only a few patients received management via transcranial approaches [9]. Nonetheless, current evidence indicates that primary orbital approaches more likely result in the recurrence of orbital cholesteatomas [9]. Furthermore, traditional orbitotomies, which often imply the removal of extensive facial bone flaps, entail the risk of postoperative infections and lesioning of important nervous structures of the face, as well as inevitable aesthetic consequences.

The pterional approach has been widely employed in neurosurgery since it's developed by Yasargil in 1975 [6]. Although initially designed to expose the anterior and medial skull base, the Sylvian fissure, the cerebral arterial circle, the basal cisterna, and the interpeduncular region, it also provides an excellent approach to the orbit [7, 8]. This is because of the ample anatomical relation of the pterion with the superolateral orbital wall, which is mainly formed by the greater wing of the sphenoid bone. Such a relationship facilitates direct access to the orbit with no need for dura matter opening nor excessive retraction of the temporalis muscle, limiting the risk of vascular and nerve injury. Moreover, lateral, posterior, and intradural extension of the pterional approach can be performed according to intraoperative requirements with a minimal amount of bony removal [8]. This facilitates the approach to lesions invading adjacent segments of the dura mater, which otherwise would be difficult to achieve by using other orbital approaches, as occurred in the case described here. These technical advantages, together with the fact that the hair covers the skin incision of the pterional craniotomy, lead to better postoperative aesthetic outcomes and constitute the rationale for the usage of such technique over other orbital approaches for the total resection of complex tumors.

Despite such benefits, the pterional approach has been rarely used in the context of orbital tumors. Previous reports have demonstrated the low operative morbidity of this approach in the management of different types of orbital neoplasia, including lacrimal, neurogenic, and metastatic tumors [8]. However, only a few cases of cholesteatomas and cholesterol granulomas have been approached by this technique [9]. In this context, the current report adds to evidence of the low occurrence of postoperative complications after the pterional approach to orbital tumors [8], as our patient showed no evidence of wound infection, cerebrospinal fluid fistula, iatrogenic enophthalmos, and exophthalmos. In addition, the patient presented a spectacular recuperation of the motor and sensorial functioning of the affected eye, despite the chronic nature of his condition. Notably, a major finding of our report is the absence of tumor recurrence, which remarks the great performance of this procedure to allow complete erase of orbital neoplasia. Finally, the current description illustrates that the reachability of the pterional approach is wider than originally described, spanning to the level of the orbital floor. However, it is important to mention that the pterional approach is not useful for orbital tumors arising at the nasal orbital wall. Despite this, our commendable clinical outcomes provide valuable evidence favoring the use of the pterional craniotomy for cholesteatomas and other extensive tumors of the superolateral orbital wall.

In conclusion, our report demonstrates that the pterional craniotomy is a safe procedure that offers advantages over primary orbital approaches for the surgical resection of orbital cholesteatomas with a low postoperative morbidity and acceptable aesthetic outcomes.

## Data Availability

The clinical data from the case presented here are available from the corresponding author on reasonable request.

## Abbreviations

MRI: Magnetic resonance imaging
MRI: Magnetic resonance imaging
